# Morphological Characterization of *Nicotiana tabacum* Inflorescences and Chemical-Functional Analysis of Extracts Obtained from Its Powder by Using Green Solvents (NaDESs)

**DOI:** 10.3390/plants12071554

**Published:** 2023-04-04

**Authors:** Mariana Leal, María Alejandra Moreno, Patricia Liliana Albornoz, María Inés Mercado, Iris Catiana Zampini, María Inés Isla

**Affiliations:** 1 Instituto de Bioprospección y Fisiología Vegetal (INBIOFIV), CONICET-Universidad Nacional de Tucumán (UNT), San Miguel de Tucumán T4000, Argentina; 2 Facultad de Ciencias Naturales e IML, Universidad Nacional de Tucumán (UNT), San Miguel de Tucumán T4000, Argentina; 3 Biolates Network for Sustainable Use of Ibero-American Vegetable Biomass Resources in Cosmetics (Biolates CYTED), San Miguel de Tucumán T4000, Argentina; 4 Instituto de Morfología Vegetal, Fundación M. Lillo, Miguel Lillo 251, San Miguel de Tucumán T4000, Argentina

**Keywords:** tobacco, pre-harvest waste, inflorescences, green solvent, NaDES, phenolic compounds, alkaloids, antioxidant activity, cosmetics

## Abstract

The production of smokeable tobacco for use in cigarettes is characterized by the production of pre-harvest and post-harvest waste, with ensuing undesirable effects on the environment. The inflorescences of tobacco after blunting, deflowering, or topping are considered pre-harvest waste and left in the field. Using green and ecofriendly solvents such as Natural deep eutectic solvents (NaDESs), these wastes could be used to obtain antioxidant molecules of interest in cosmetics. Taking into account its potential as plant matrix to obtain metabolites of commercial interest, tobacco inflorescences and inflorescence powders of different particle sizes were characterized by optic and electronic microscopy. Thus, the powdered inflorescences were extracted with four conventional solvents, i.e., distilled water (DW), acetone: distilled water (AW), ethanol 70° (EW), methanol (Me), and five NaDESs, i.e., lactic acid: sucrose (LAS), lactic acid: sucrose: distilled water (SALA), fructose: glucose: sucrose: distilled water (FGS), choline chloride: urea: distilled water (CU), and citric acid: propylene glycol (CAP). Among the tested NADESs, SALA was the most promising solvent; higher extraction yields of total phenolic compound (3420.0 ± 9.4 µg GAE/mL) than conventional solvents were attained and it was the only selective solvent to phenolics. CU was the best solvent for flavonoids and alkaloids extraction (215.3 ± 3.2 µg QE/mL and 392.3 ± 8.0 µg ACE/mL, respectively). All extracts showed antioxidant activity. A heatmap with dendrogram and main component analysis showed that acid-based NaDESs are grouped together, this group being the one with the best performance in H_2_O_2_ scavenging. The extracts obtained with green solvents could be used directly in cosmetic formulations as antioxidant ingredients because both tobacco flower oil and flower extracts are listed in the cosmetic ingredients database as non-toxic products. Additionally, the demand for sustainable ecological cosmetics is growing. In this sense, NaDESs represent an opportunity to develop innovative extracts with unique phytochemical fingerprints and biological activities.

## 1. Introduction

**Figure 1:**
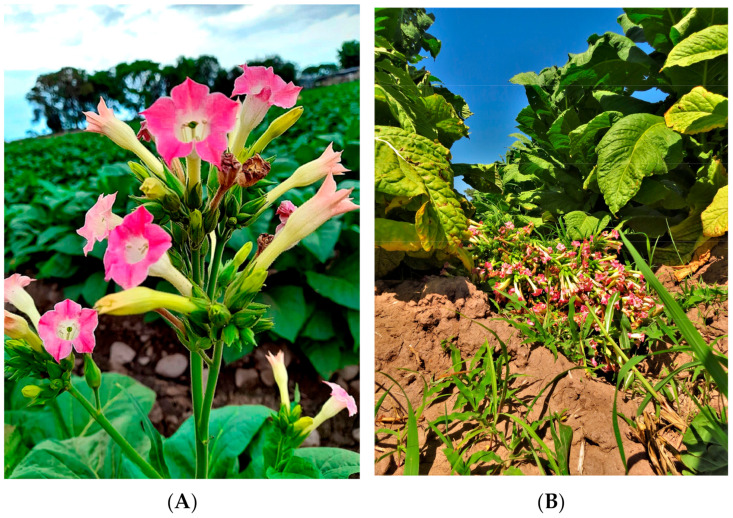
*Nicotiana tabacum* var. Virginia. (**A**) Inflorescences. (**B**) General aspect of pre-harvest waste produced during the blunt (inflorescence and apical leaves) and thrown in the field (Perico, Jujuy).

*Nicotiana tabacum* L., (commonly known as tobacco) is one of the most economically important industrial crops worldwide and the main species for the commercial production of smokeable tobacco [1,2]. Approximately 6 trillion cigarettes are consumed worldwide each year and the total tobacco production is approximately 6.68 million metric tons [2]. Accordingly, such a big production leads to trillions of pre- and post-harvest wastes during the manufacture of tobacco cigarettes. Before the harvest of broad-leaf tobacco, plants are blunted, deflowered, or topped, i.e., flower buds and some of the upper leaves are removed in order to stimulate the development of the remaining lower leaves, thus leaving discarded flowers or inflorescences on the field (Figure 1). In Argentina, the plant density of tobacco var. Virginia (light tobacco) is around 20,000 plants ha^−1^ with a total pre-harvest waste biomass (inflorescence) of 2000 kg·ha^−1^. In a world where economies tend to be circular, with “zero waste” policies implemented, such a loss is unthinkable. Thus, the inflorescences of tobacco which represent an unutilized biomass from the agricultural production of cigarette tobacco could be used, for example, for cosmetic or pharmaceutical purposes.

Previous chemical studies revealed that tobacco flowers contained polysaccharides [3], sterols [4], terpenoids compounds [5,6,7,8,9,10], alkaloids [10], and phenolic compounds, mainly flavonols like rutin, quecetin, isoquercitrin, kaempferol 3-glucoside, quercetin 3–mono, 3,3′-dimethyl ether, and kaempferol 3-rutinoside-7-glucoside [11,12,13,14], and anthocyanins such as cyanidin, delphinidin, peonidin, and petunidin [15,16,17]. Furthermore, although numerous compounds have been identified in tobacco flowers, studies on biological activities for potential industrial uses are scarce, i.e., antimicrobial, antioxidative, and antitumoral, not to mention their use as pesticides were described only for terpenoids compounds obtained from tobacco flowers [3,7,8,9,15,16,17]. However, to the best of our knowledge, no studies on the biological activities of the phenolic compounds present in the inflorescences have been issued until now.

Within this context, inflorescences could be further used as a subproduct to obtain bioactive metabolites for phytopharmacy, phytocosmetic, and perfumery uses. Tobacco flower oil and different flower extracts are referenced in the Cosmetic Ingredient Database, CosIng [18], as cosmetic ingredients with perfuming functions. These ingredients are not listed in Annex II (banned cosmetic ingredients) or Annex III (restricted cosmetic ingredients) of Regulation No 1223/2009 (the Cosmetics Regulation) [19], except nicotine as an individual substance. Tobacco flowers have recently been used to obtain an aromatic cosmetic product, a concrete. The product was obtained by extraction with non-polar organic solvents, further concentrated by complete removal of the solvent, and was chemically characterized [10]. Previously, gels containing phytosomes were developed as a delivery system for *Nicotiana tabacum* var. Virginia leaf extracts [20]. Furthermore, a cosmetic gel incorporating resinoid obtained with ethanol from leaves of tobacco var. Virginia, Burley, and Oriental as an active ingredient was obtained [21]. More studies on tobacco flowers’ bioactive compounds are necessary to assert the potential of currently discarded plant material in the field of cosmetics.

To date, organic solvents such as ethanol, anhydrous ethanol, methanol, isopropyl alcohol, ethyl acetate, acetone, methylene chloride, and n-hexane have been used to extract different compounds from tobacco flowers [4,5,6,7,8,9,10,11,12,13,14,15,16,17]. However, these solvents have proved to be relatively toxic and could remain not only as a residual pollutant in the extracts of flowers produced, but also as air pollutants that contribute to global warming [22,23]. Thus, using environmentally friendly materials already existing in nature can effectively help solve environmental problems such as resource shortage, pollution, and ecological destruction [24].

The use of green solvents such as Natural deep eutectic solvents (NaDESs) could overcome the limitations of traditional organic solvents. The NaDESs can be defined as eutectic mixtures formed by a combination of natural compounds, i.e., sugars, organic acids and bases, and amino acids, with specific molar ratio. They melt at a single, sharp unique temperature called the “eutectic point” (the lowest melting point for a specific composition) and is characterized by a supramolecular structure. These solvents present several advantages over traditional ones, i.e., they are easy to prepare, and represent renewable/nontoxic natural precursors, besides being low-cost and biodegradable. Because of this, they are widely employed in organic synthesis, biocatalysis, drug dissolution, and chemistry of materials [25,26,27]. As NADESs are a subclass of DES, they share similar characteristics, except that the former are based on natural components [25]. For DES, and therefore for NADESs, the choice of solvents is very important to determine their properties. Such properties include phase behavior, melting-point temperature, density, conductivity, surface tension, viscosity, and polarity. One of the advantages for the application of NaDESs as a green extraction solvent is that the properties described above can be easily adapted either by changing the selection of components or the molar ratio between the components, and also by adding water [28]. This homogenous liquid is stable once the eutectic point is reached, easing its use for the extraction of bioactive compounds from plant tissues. In particular, the extraction efficiency of phenols, terpenoids, and other compounds increased when NaDESs were employed instead of traditional solvents [29,30]. NaDESs are stable and can extract both hydrophilic and hydrophobic compounds from plant matrix [25,26,27]. These properties open a gate to the development of innovative extracts with unique phytochemical profiles and biological activities for use in pharmaceutical and/or cosmetic products [27,29].

Based on the aforementioned analysis, the present research focuses on optimizing the extraction of bioactive compounds, particularly phenolic ones, by both using conventional and green solvents (NaDESs) and assessing their antioxidant activity in cosmetic products. To the best of our knowledge, no specific studies on the advantage of NaDESs over conventional organic solvents regarding the extraction of phenolic compounds from tobacco inflorescences have been conducted so far. Five kinds of NADESs were tested for extraction and compared with methanol/HCl, EW, AW, and DW as the conventional reference solvents. The characterization of the morpho-anatomy of *N. tabacum* (var. Virginia) inflorescences and powdered inflorescences was also performed to ensure the pharmacognostic identification of the material and to avoid substitution by other plants in case commercial products are developed.

## 2. Results and Discussion

For the present study, inflorescences obtained during blunting of *Nicotiana tabacum* var. Virginia were collected in a field in Perico, Jujuy, Argentina. Tobacco inflorescences, as well as powdered ones, were used for morphological studies and microscopically characterized, respectively. The powdered inflorescence was used for the extraction of bioactive compounds by using conventional and green solvents (NaDESs) and for assessing their antioxidant activity.

### 2.1. Flowers Morphology and Anatomy

**Figure 2:**
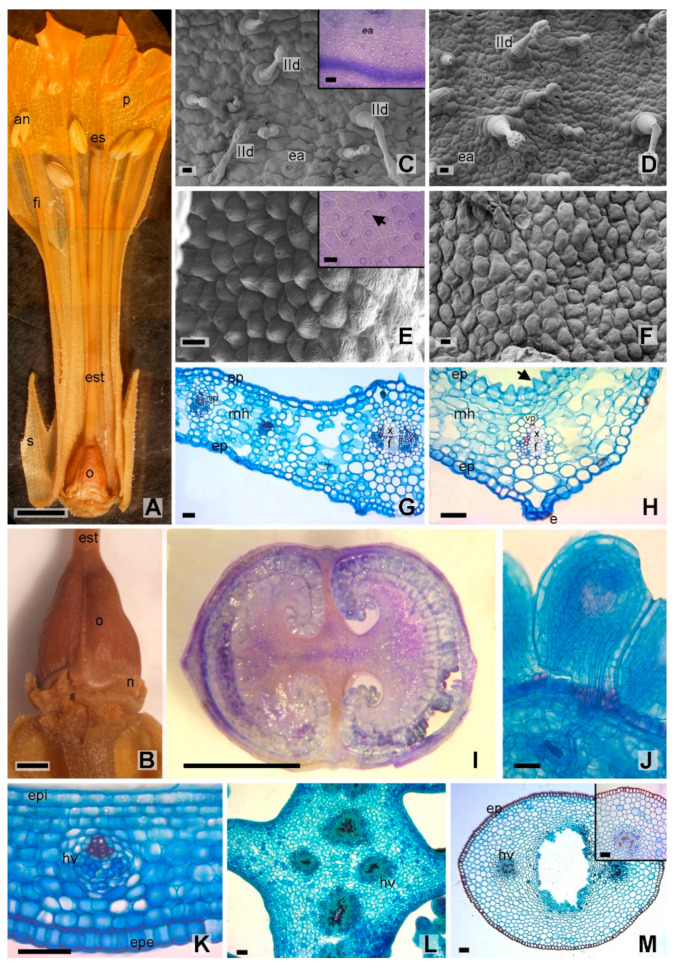
*Nicotiana tabacum* var. Virginia. Morphology and anatomy of the flower. (**A**) General appearance. (**B**) Detail of ovary and nectary. (**C**–**H**) Perianth. (**C**–**F**) Superficial view of epidermis in MEB. (**C**) Sepal, upper epidermis, insert detail in light microscope. (**D**) Sepal, lower epidermis. (**E**) Petal lobe, papillated upper epidermis, inset detail in light microscope. (**F**) Petal, lower epidermis. (**G**) Transverse section of sepal. (**H**) Transverse section of petal. (**I**–**M**) Gynoecium (**I**–**L**) Cross section of ovary. (**I**) General appearance. (**J**) Anatropous ovule. (**K**) Ovary wall. (**L**) Ovary central portion. (**M**) Cross section of the style, insert, detail of the lignified epidermis and vascular bundle. References: an, anther; e, stoma; ea, anomocytic stoma; ep, epidermis; epe, outer epidermis; epi, inner epidermis; es, stigma; est, style; f, phloem; fi, filament; arrow, papilla; hv, vascular bundle; IId, glandular trichome with uniseriate stem and multicellular head; mh, homogeneous mesophyll; n, nectary; o, ovary; p, petals; s, sepals; vp, parenchymal sheath; x, xylem. Scales: (**A**) 5 mm; (**B**,**C**,**J**) 20 µm; (**D**,**E**), 10 µm; (**F**,**G**,**K**–**M**), 50 µm; (**H**,**I**), 2 mm.

The flower of *Nicotiana tabacum* var. Virginia is hermaphrodite, tubular, infundibuliform-rotate to campanulate. The perianth is welded and pubescent, mainly on the abaxial (exterior) surface. The calyx is synsepalous, formed by 5 triangular sepals. The gamopetalous corolla with 5 white-pinkish petals extends upwards, forming a throat with a pentagonal lobed limb (Figure 2A). The superior ovary is supported on a nectariferous disc (Figure 2B). The style is single and massive, extending along the corolla tube and ending in a bilobed stigma. The stamens (5) are inserted at the base of the throat of the corolla, and the anthers are dorsifixed (Figure 2A). Similar observations were previously made by other authors for this species in other cultivars [31,32,33].

#### 2.1.1. Perianth Anatomy

In superficial view, both epidermises of the sepals showed rectangular to isodiametric cells with slightly striated cuticles in the proximity of the stomata. Epidermal cells presented straight to sinuous anticlinal walls. The stomata are anomocytic, slightly raised in relation to the rest of the epidermal cells (Figure 2C,D). The trichomes distributed on both epidermal surfaces were simple and glandular type I, IIa, IIb, IIc and IId, as previously described by Leal et al. [29], for the tobacco leaves var. Virginia.

The adaxial epidermis of the petal lobes showed distinctive papillose cells, isodiametric with slightly lobed anticlinal walls, and smooth cuticle, whereas in the abaxial epidermis, the cells ranged from rectangular to isodiametric with straight to sinuous anticlinal walls, with a slightly striated cuticle in the proximity of the stomata (Figure 2E,F), and glandular trichomes of type IIb, IIc and IId.

In cross section, sepals and petals presented one layered epidermis, homogeneous parenchymal mesophyll with air chambers, and collateral vascular bundles with parenchymal sheaths (Figure 2G,H).

#### 2.1.2. Gynoecium Anatomy

The ovary was conical in shape, supported by a nectary. In superficial view, the external wall of the ovary presented isodiametric cells with a smooth cuticle. In cross section, the ovary was bicarpelar, bilocular with axillary placentation (Figure 2I). As previouly described by Chang and Sun [32], the ovules were anatropous, bitegumented (Figure 2J). Both epidermis in the ovary wall were one layered; homogeneous parenchymal mesophyll of 5–8 layers, prismatic crystals of calcium oxalate and collateral vascular bundles were also observed (Figure 2K). In the central portion four ectophloic ventral vascular bundles were arranged in a cross (Figure 2L).

**Figure 3:**
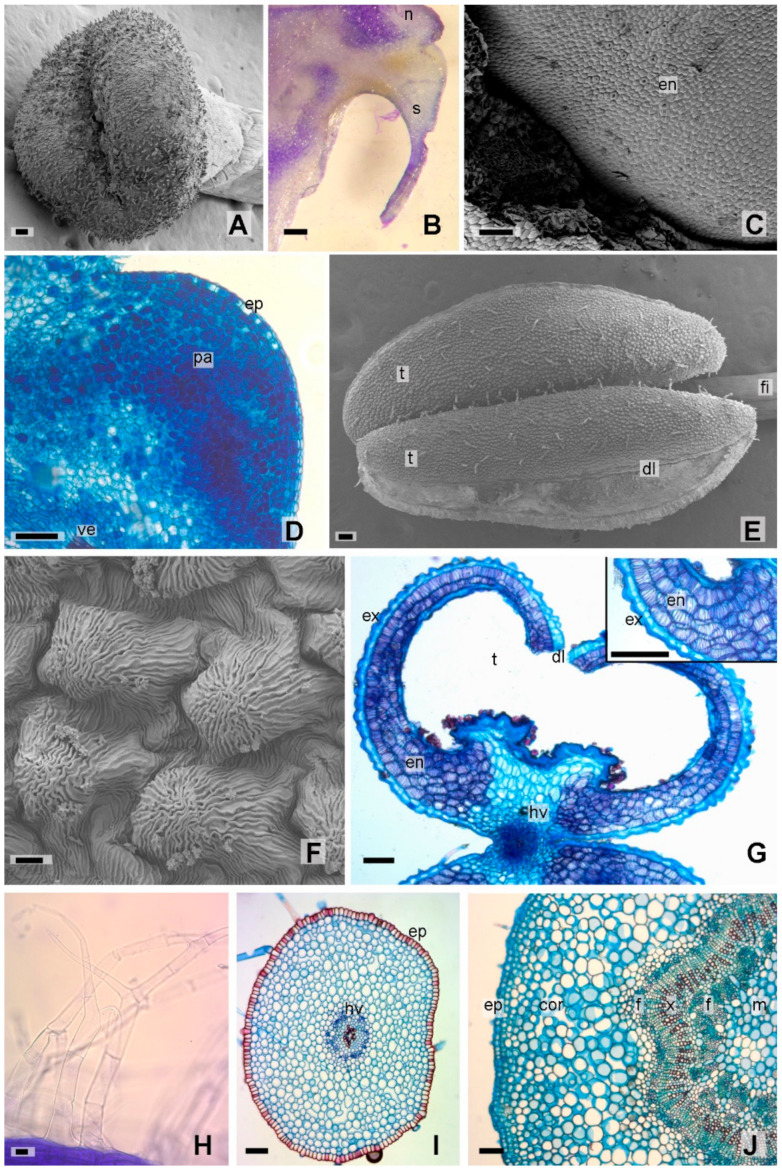
*Nicotiana tabacum* var. Virginia. (**A**–**D**) Gynoecium (**A**) Bilobed stigma in SEM. (**B**–**D**) Floral nectary. (**B**) Cross section. (**C**) Superficial view of the nectary in SEM. (**D**) Detail of the nectary. (**E**–**I**) Androecium. (**E**) Anther and filament in SEM. (**F**) Detail of the exotecia of the anther in SEM. (**G**) Cross section of anther, insert detail of exo and endothecium. (**H**) Branched trichomes of the filaments. (**I**) Cross section of the filament. (**J**) Cross section of the floral pedicel. References: cor, cortex; dl, longitudinal dehiscence; en, nectariferous stoma; ep, epidermis; f, phloem; fi, filament; hv, vascular bundle; m, medulla; n, nectary; pa, parenchyma; s, know it; t, teak; ve, external vascularity; x, xylem. Scales: (**A**–**E**,**G**,**I**–**J**) 100 µm; (**F**,**H**) 10 µm.

In cross section, the middle sector of the style presented a subcircular shape, with a few simple (pluricellular uniseriate) trichomes and glandular trichomes type I, IIb, and IId. The epidermis was one layered, made up of typical cells and fibers with a lignified wall, with a smooth and thick cuticle. Internally, the ground parenchyma was formed by 8 to 20 cell layers, in which two collateral vascular bundles were immersed, facing each other and separated in the central sector by the transmission tissue (rich in starch) (Figure 2M). As described by Stabentheiner et al. [34], the stigma was bilobed with a central groove and stigmatic hairs on its surface (Figure 3A).

#### 2.1.3. Floral Nectary Anatomy

The nectary was classified as automorphic due to the protrusion of the nectariferous tissue [34] with a more or less accentuated development in the commissural sectors. It was yellowish-green in colour, lighter than the rest of the ovary (Figure 2B). In superficial view, epidermal cells were isodiametric with a smooth cuticle and secretory stomata (Figure 3C). The cross section presented a single-layered epidermis, compact homogeneous ground parenchyma with dense cytoplasm, and vascularization external to the nectary (Figure 3B,D). Similar traits were described by other authors [33,35,36,37]; in fact, Cocucci and Galetto [38] pointed out that tobacco nectary dimentions and features seem to be associated with the sphingophilic × chireptophilic inheritance of *Nicotiana* nectaries and Solanaceae in general.

#### 2.1.4. Androecium Anatomy

In the male cycle, the androecium was inserted, and the anthers were lacriform with longitudinal dehiscence (Figure 3E). On superficial view, the cells were papillose, isodiametric, with a strongly striated cuticle and simple and glandular trichomes of types IIb, IIc, and IId (Figure 3E,F). In cross section, they presented two thecae joined by connective tissue, in which, a vascular bundle of xylem surrounded by phloem (ectophloic) could be seen (Figure 3G). Each theca had two pollen sacs with tricolporated pollen grains, as described by Chang and Sun [32]. The exothecium was single-layered, while the subepidermal endothecium presented 2–4 layers of parenchymal cells with lignified secondary thickenings in a helical and Y-shaped form. Internally, the tapetum was formed by thin-walled isodiametric parenchymal cells (Figure 3G). The filament, in superficial view, presented rectangular cells with smooth cuticle. Towards the base of the filament abundant simple, multicellular, uniseriate, dichotomously branched trichomes (Figure 3H), and glandular trichomes type IIa and IId were observed. In cross section, it was subcircular with, single-layered epidermis formed by thick-walled, U-shaped (especially towards the outer surface) lignified cells; internally, 12–15 parenchyma layers with isodiametric cells and intercellular air spaces were observed. In the central position, a single vascular bundle of xylem surrounded by phloem was evident (Figure 3I). Calcium oxalate crystals were found in the inner layers of the parenchyma, close to the phloem.

#### 2.1.5. Flower Pedicel Anatomy

In cross section, the floral pedicel presented subcircular shape, with a single-layered epidermis and smooth cuticle. The stomata were slightly elevated in relation to the rest of the epidermal cells. The trichomes were similar to the types described for the leaves by Leal et al. [29]. The subepidermal collenchyma was formed by 2–3 layers, followed by 10–12 layers of cortical parenchyma with intercellular air spaces; lastly, an amyliferous perivascular sheath was observed internally. The vascular system showed an early secondary growth due to the continuity of the cambium; xylem vessels towards the internal phloem (unusual growth), and a parenchymal pith with starch grains were evident in an amphiphloic stele (Figure 3J).

### 2.2. Tobacco Inflorescence Powder Characterization

**Figure 4:**
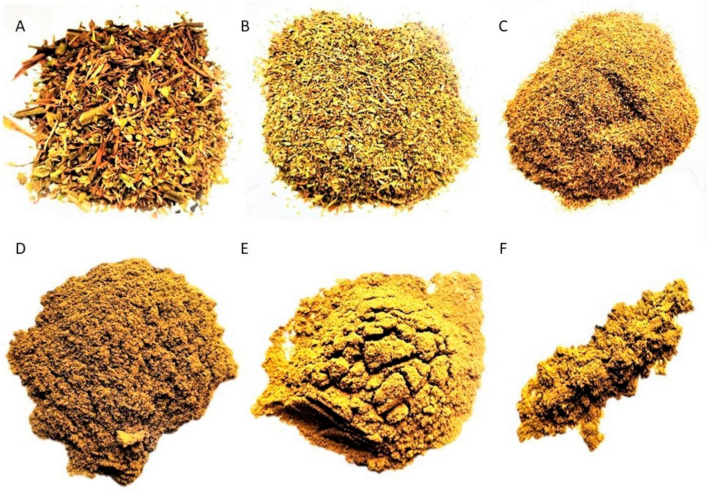
*Nicotiana tabacum* var. Virginia sieved fractions from ground inflorescence. (**A**) 840 µm. (**B**) 500 µm. (**C**) 149 µm. (**D**) 105 µm. (**E**) 74 µm. (**F**) less than 74 µm.

**Figure 5:**
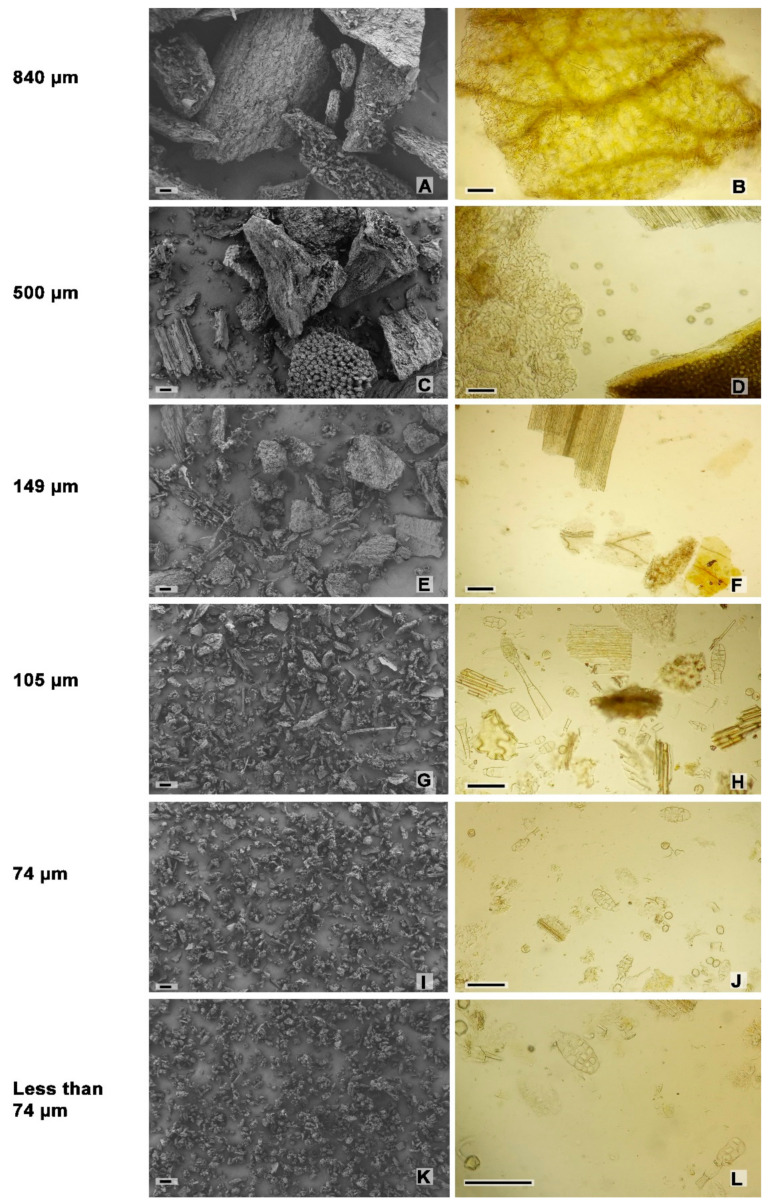
*Nicotiana tabacum* var. Virginia. Sieved fractions from ground flowers. (**A**,**C**,**E**,**G**,**I**,**K**) SEM. (**B**,**D**,**F**,**H**,**J**,**L**) Optic microscopy. (**A**,**B**) 840 µm. (**C**,**D**) 500 µm. (**E**,**F**) 149 µm. (**G**,**H**) 105 µm. (**I**,**J**) 74 µm. (**K**,**L**) less than 74 µm. Scales: (**A**–**L**) 100 µm.

The tobacco dry inflorescences were ground to powder in order to reduce the size of the particles so as to favor the subsequent processes of extraction of bioactives (Figure 4). Then, the particles size was determined and a microscopical analysis of powdered inflorescences was performed. Microscopical analyses under scanning electron and optical microscopy of the sieved powder showed particles of different sizes and tissue composition. The particle sizes range between <74 µm to 840 µm (Table 1, Figure 4). The 840 µm, 500 µm and 149 µm fractions presented complete fragments of floral parts, such as sepals, petals, anthers filled with pollen grains, and filaments, with their characteristic structures. The total yield of particles with mesh size between 149 to 840 µm was about 93.53%. Whereas 105 µm, 74 µm, and less than 74 µm fractions exhibited mainly trichomes, trichome heads, pollen grains, parenchyma cells of the mesophyll, cuticle fragments, epidermal remains, and occasional fragments of xylem vessels (Figure 5).

### 2.3. Phytochemical Characterization by Conventional and Non-Conventional Solvents

Grinding tobacco dry inflorescences reduced the particle sizes to micron scale, allowing an increased specific surface area for extraction trials. Five NaDESs, LAS, SALA, CAP, FGS, and CU (Table 2) were used to extract different chemical components from ground inflorescences. The efficiency of the extraction was compared to that of the standard solvents AW, EW, and DW. The content of total phenolic compounds (TPC) of *N. tabacum* inflorescences powder var. Virginia ranged from 1326 to 2711 µg GAE/mL in the extraction with DW, AW, and EW, conventional solvents; with NaDESs, the values ranged from 1305 to 3420 µg GAE/mL (Table 3). The content of TPC in inflorescences was higher than in pre-harvest tobacco leaf waste [29]. SALA was more effective and selective in TPC extraction and could be considered the best solvent (Table 3), followed by CU, LAS, and EW, which showed the same behavior. FGS and AW showed the same extraction potency. It was lower than CU, LAS, and EW. In our study, the NaDESs SALA, CU, and LAS were the most promising solvents, attaining higher extraction yields of TPC. SALA was the most selective to TPC extraction, since it extracts 10 times fewer alkaloids than conventional solvents such as alcohol and acetone; thus, it could have a greater potential for use in ecofriendly low-alkaloid products. Considering that planting density of tobacco var. Virginia is around 20,000 plants ha^−1^ and produces a total pre-harvest waste biomass (inflorescence) of 2000 kg·ha^−1^, the phenolic compound yield extracted with SALA (11.8 Kg GAE·ha^−1^) could be considered an option for the sustainable use of inflorescence.

The extraction yield of flavonoids, a type of phenolic compound, was analysed. The total content of flavonoid compounds showed values between 122 and 204 µg QE/mL. CU, a NaDES of choline chloride as the hydrogen bond acceptor in combination with urea as hydrogen bond donor, was the best extraction solvent for flavonoids, with the same yield as conventional solvent such as EW. The order of efficiency in the extraction of the TF from the powdered flowers was as follows CU = EW > AW = DW > FGS = SALA > LAS > CAP (Table 3). The efficacy of CU was previously reported for flavonoids, i.e., quercetin and kaempferol [39]. Previously, the presence of flavonoids was reported in corolla or petals and calyxes or sepals of tobacco flower, using organic solvents for its extraction, namely isopropyl alcohol-water (1:1, *v*/*v*), followed by solvents which progressively increased in alcohol content [12].

In previous studies, anthocyanin contents were regarded as the major factor which gave the flower the pink or light red colour in transgenic tobacco [16,40,41]. The flower colour is an important trait in many plants and flower coloration has been one of the hotspots in biological studies. Previously, the presence of anthocyanins was also reported in tobacco leaves [15]. As far as we know, this is the first report on the presence of anthocyanin in Virginia variety tobacco flowers. The pigments were extracted mainly with methanol/HCl 1%, followed by SALA, AW, LAS, and DW (Table 3). It is important to note that SALA also allows the selective extraction of TPC from the inflorescence powder. Other authors reported that organic acid-based NaDESs had the highest affinity in the H-bond acceptor region and are more polar than water, whereas sugar and polyalcohol-based NaDESs display low polarities, close to that of methanol [25]. Given that anthocyanins are polar molecules that are better solubilised in water than in non-polar solvents and that pH has great importance for anthocyanin extraction and stability, it was not surprising that, among the NaDESs employed, the acidic and most polar ones, such as SALA and LAS, resulted in a high concentration of total anthocyanins [42]. The flavylium ion is stable under acidic conditions, but when the pH is raised to weakly acidic mediums between pH 2 and 4, the structures of the bonds around the flavylium ion change to form the blue quinoidal species. At pHs between 5 and 6, the colourless carbinol pseudobase and chalcone are predominant, while at pHs > 7, the anthocyanins are degraded [42]. NaDESs were assayed for the recovery of anthocyanins from by-products derived from red grapes [43,44,45], blueberries [46], and jaboticaba fruit [47]. All the studies performed revealed that the molar ratio among the components of the eutectic mixture, the amount of added water, and the solid-to-liquid ratio had a marked impact on the extractive capacity of NaDESs. In general, an increase in the water content in the mixture caused a decrease in its viscosity, a fact which improved the mass transfer rate between the solid and the liquid phase and thus facilitated the extractability of anthocyanins from the matrix.

The use of NaDES for the extraction of alkaloids is still very limited. Alkaloids derived from morphinane, protoberberine, bisbenzylisoquinoline, indole, and quinolizidine were extracted by using choline chloride with organic acid or urea and water [48]. In another recent work, different NaDESs were used for the extraction of alkaloids from Amarillydaceae [49]. Five NaDESs and conventional solvents (DW, AW, and EW) were tested in the present work in order to extract tobacco inflorescence alkaloids. AW and EW were the best solvents for the extraction of this type of metabolites. According to our results, 1 g of inflorescence powder would have about 6000 µg alkaloid, which represents 50% of that reported by other authors for leaves [50]. Among the NaDESs, the most effective was CU, which extracts about 60% of the alkaloids present in inflorescence compared to conventional solvents [51]. Recently, Leal et al. [29] reported that the best solvent for alkaloid extraction from tobacco leaves was also CU.

### 2.4. Antioxidant Activity

Plant bioactive extracts are promising not only for the pharmaceutical and cosmetic industries, but also for the food industry. Plant extracts with antioxidant capacity could be used to eliminate free radicals, which are responsible for cell damage, avoiding the development of numerous pathologies associated with oxidative stress. Both the extraction method (maceration, ultrasound, supercritical fluids, microwave-assisted extraction, high pressure) as extraction solvents (conventional and non-conventional) of bioactives from plant material play a key role in the chemical composition of extracts. The solvent: biomass rate, the size particle of powder, as well as the conditions of extraction (temperature and pressure) are also of crucial importance for the chemical and biological quality of plant extracts [4,29]. Different reports showed that tobacco leaves organic extracts enriched in polyphenolic and terpenoid compounds scavenge free radicals [50,51,52]. Similarly, the antioxidant activity of *N. tabacum* roots hexanic extract [53] and flowers organic extracts [3,4,7,8] was demonstrated. However, as mentioned above, organic solvents pollute the environment when they evaporate, leaving behind toxic residues. The NaDESs have emerged as a promising, eco-friendly alternative to petrochemicals to dissolve plant metabolites when used in food, cosmetics, and phytotherapic products. Recent findings showed that polyphenols extracted with NaDESs from leaf waste of tobacco scavenge free radicals [29]. In the present work, the antioxidant activity of tobacco inflorescence extracts obtained with NaDESs and other conventional solvents were compared for the first time. The SC_50_ values were defined as the concentration of antioxidant needed to reduce 50% of the initial free radical. A lower SC_50_ value entails a higher antiradical activity of the extract. The highest antioxidant activity on ABTS scavenging was observed for inflorescence extracts obtained with the green solvents FGS and CU (Table 4). Among the conventional solvents, the EW extract was the most active, followed by AW (SC_50_ = 3.6 and 8.0 µg GAE/mL, respectively). The H_2_O_2_ scavenging activity was higher in the extracts obtained with CAP, followed by LAS and SALA (Table 4). This activity was significantly higher than that of the extracts obtained with conventional solvents and CU and FGS. It is interesting to note that the SC_50_ values could not be explained simply by the content of total phenolic compounds determined; this suggests that some other factors influence the overall antioxidant activities of extract, i.e., the phenolic type. The antioxidant activity of the each NaDESs itself was also determined because some NaDES-forming compounds, such as organic acids, are known antioxidants [54]. The experimental data evinced that NaDESs shows an excellent ability for the extraction of antioxidant compounds of tobacco inflorescence powder and are, consequently, an opportunity for a more sustainable extraction of antioxidant ingredients for the cosmetic industry, as they could be included in gels, lotions, and creams, among other toiletries.

### 2.5. Correlation between the Total Phenolic Compounds, Total Flavonoid, Total Alkaloid and Total Anthocyanin Content, and Antioxidant Activities

Pearson’s correlation coefficient was applied to evaluate the relationship between the antioxidant activity, i.e., ABTS and H_2_O_2_, and secondary metabolite content, including total phenolic content (TPC), total flavonoids (TF), total alkaloids (TA), and total anthocyanins (TAC), as shown in Table 5 Significant positive correlations between TPC, TF, and TA with H_2_O_2_ scavenging activity were found, with Pearson’s correlation coefficients r = 0.09, r = 0.77, and r = 0.87, respectively (*p* < 0.05). Furthermore, ABTS radical cation scavenging activity showed a significantly high positive correlation with TAC (Pearson’s correlation coefficient r = 0.73, *p* < 0.05). A recent study suggested that high anthocyanin content in transgenic tobacco allows the plants to tolerate chilling stress with relatively low ROS accumulation [55]. However, this is the first report for ABTS radical cation scavenging activity and its relationship with the tobacco anthocyanin components.

**Figure 6:**
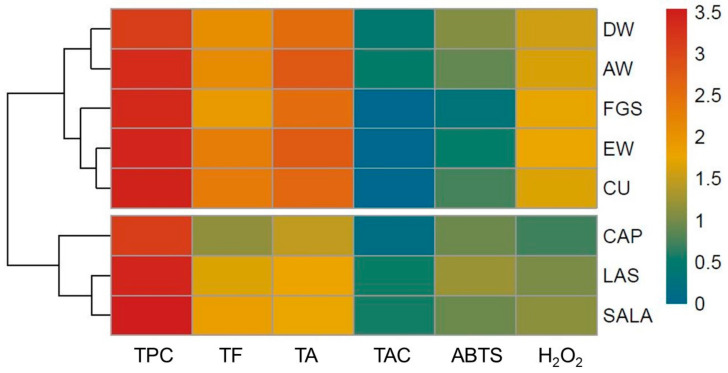
Heatmap and dendrogram of the total phenolic content (TPC), total flavonoid (TF), total alkaloids (TA), total anthocyanin (TAC), and antioxidant activity (ABTS, and H_2_O_2_ scavenging activity) of extracts from tobacco inflorescence waste obtained with conventional and non-conventional solvents. DW: distilled water; EW: ethanol 70°; AW: acetone:water; NaDESs: LAS, CAP, FGS, SALA, CU.

**Figure 7:**
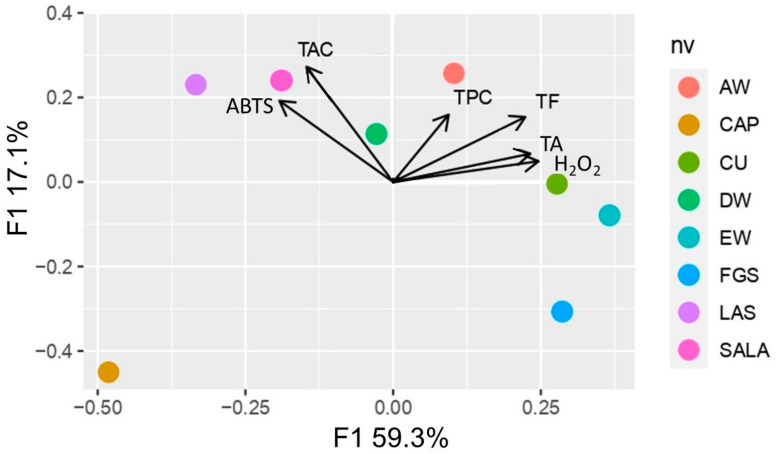
Main component analysis of the total phenolic content (TPC), total flavonoid (TF), total alkaloids (TA), total anthocyanin (TAC), and antioxidant activity (ABTS, and H_2_O_2_ scavenging activity) of extracts from tobacco inflorescence waste obtained with conventional and non-conventional solvents. DW: distilled water; EW: ethanol 70°; AW: acetone:water; NaDESs: LAS, CAP, FGS, SALA, CU.

To provide an overview of the grouping of the extracts obtained with different solvents, a heatmap with dendrogram was used. The effect size measure is represented by the color intensity. The heatmap clusters similar rows and similar columns together, with their similarity represented by a dendrogram. Considering Figure 6, it is possible to separate the samples into two groups. The first group, acid-based NaDES, is characterized by both the best performance in hydrogen peroxide (H_2_O_2_) scavenging activity and high efficiency in TPC extraction with lowest TA content. The second group (CU, EW, FGS, AW, and DW) is characterized by the highest concentration of TF and TA, and also the best ABTS scavenging activity. The main component analysis that sets together SALA, LAS, and CAP (Figure 7) supports the heatmap analysis.

The main component analysis (Figure 7) shows a 76.4% total variability, explained by factors F1 and F2. F1 explains 59.3% of the variability and is positively influenced by H_2_O_2_ and TA. F2 explains 17.1% of the variability and is influenced by TPC. This component clearly separates SALA, CAP, LAS, and DW from AW, EW, CU, and FGS. The groups formed are similar to those mentioned above. The distance between two assays represents their proximity level; the closer the two vectors are, the more significant their correlation is. For instance, the distance of both ABTS scavenging activity and TAC was very short; thus, it contained significant positive correlations with similar distances to those between H_2_O_2_ scavenging activity with TF and TA.

## 3. Materials and Methods

### 3.1. Plant Material

Inflorescences of *Nicotiana tabacum* Var. Virginia discarded during pre-harvest of tobacco production were collected from a crop field in the locality of Perico, Jujuy, Argentina (26°18′ S, 65°37′ W, 1700 m.a.s.l.). The samples were dried at 60 °C, up to constant weight in a forced-air stove (Tecnodalvo, TDSI F70, Santa Fé, Argentina) and conserved in vacuum sealed bags until use. Fresh flowers for histological assays were collected from 5 random individuals and preserved in FAA (acetic acid, formalin, water and alcohol 1:2:7:10) for both optical and scanning electron microscopy (SEM) studies.

### 3.2. Powder Obtention by Milling

The dried inflorescences were grounded in a Helix mill (Metvisa^®^, Mod MP-200-Power ½ HP-0.75 Kw. Brusque, Brazil) to obtain inflorescence powder. The size particle, or granulometry, was determined by vibrating screen sieving (Zonytest, Buenos Aire, Argentina) with 840, 500, 149, 105, and 74 µm sieves.

### 3.3. Histological Analysis

#### 3.3.1. Light Microscopy

The characterization of the flower epidermis and analysis of the fragments in the powders was made by using the diaphanizations technique, according to Dizeo de Strittmater [56]. The analysis of the structures was carried out through cross sections, obtained by mounting the different flower parts on a dental wax support and subsequently sectioning with a Microm HM315 rotary microtome (GMI Inc., Ramsey, MN, USA) [57]. The diaphanized tissues were stained with crystal violet and directly observed under the microscope and stained with crystal violet, in the case of powder. The sections were cleared with 50% sodium hypochlorite, washed with distilled water, stained with astra blue-safranin, and mounted in water-glycerin (1:1) [58]. Monitoring was made with a stereoscopic microscope (Olympus SZX7, Tokyo, Japan) and an optical microscope (Carl Zeiss Axiostar Plus, Göttingen, Germany) attached to a photographic camera (Carl Zeiss AxioCam ERc 5s, Oberkochen, Germany).

#### 3.3.2. Scanning Electron Microscopy

For scanning electron microscopy (SEM), samples previously fixed in FAA were dehydrated with a series of alcohols and acetone and submitted to dryness critical point with liquid CO_2_. Dry fractions of inflorescence powders were directly attached to SEM stubs by using a carbon double-adhesive disc. Pretreated samples of flower parts and dry fractions of the powders were coated with gold-palladium by using a Fine Coat Ion Sputter JEOL JFC-1100. A scanning electron microscope (Carl Zeiss Supra 55VP, Oberkochen, Germany) from the Integral Center for Electron Microscopy (CIME, UNT-CONICET) was used.

### 3.4. NaDESs Preparation

The NaDESs were prepared by mixing the components in the appropriate mole ratios (Table 2), as follows: FGS, CU, and CAP were prepared according to Dai et al. [25], Delso et al. [59], and Grozdanova et al. [60], respectively. LAS and SALA were prepared according to Macchioni et al. [61] and Doldolova et al. [62], respectively. They were soaked in a water bath at 40 °C; a stable transparent liquid was obtained after stirring for 20 min. The reagents were acquired in Cicarelli, Santa Fe, Argentina (ethanol, acetone, sucrose, fructose), Anedra, Bs As, Argentina (glucose, citric acid, urea), Biopack, Bs As, Argentina (propylene glycol), Sigma-Aldrich, Beijing, China (choline chloride 98.9% purity and lactic acid, D,L-lactic acid 91.4% purity).

### 3.5. Inflorescence Powder Extraction

Inflorescence powder was extracted by maceration in each solvent at a ratio 1:10 (w:v) for 30 min shaking at 100 rpm at 25 °C. The extracts were filtered and kept at −20 °C until use. The same procedure was used with all solvents: ethanol 70°, 1:10 (EW), distilled water (DW), acetone: distilled water, 1:2 (AW), methanol:HCl 1% (Me:HCl 1%), and NaDESs (LAS, SALA, CAP, FGS, CU).

### 3.6. Determination of Chemical Composition

#### 3.6.1. Total Polyphenols and Flavonoids Content Determination

The total phenolic compound content was determined by using Folin–Ciocalteu reagent [63]. The blue color developed was read at 765 nm in UV/visible spectrophotometer (Jasco v-630, Thermo Fisher Scientific, Tokyo, Japan). Total flavonoids were estimated by using the Woisky and Salatino method using AlCl_3_ reagent [64]. The conventional and non-conventional solvent controls were performed in each determination to scan any possible interference. The determinations were performed in triplicate and the results were expressed as μg of gallic acid equivalent (GAE) per milliliter (μg GAE/mL) and quercetin equivalents (QE) per milliliter (μg QE/mL) for polyphenols and flavonoids, respectively. Folin–Ciocalteu reagent, phenol reagent, AlCl_3_, gallic acid, and quercetin were acquired at Sigma Aldrich, St. Louis, USA. The extraction yield in each solvent was determined as the amount of chemical component per mL of extract.

#### 3.6.2. Total Anthocyanins Content Determination

Dried leaves (1 g) were extracted with 1% HCl in methanol to determine total anthocyanins. Anthocyanin content was also measured in each extractive solutions (EW, DW, AW, NaDESs), according to Lee et al. [65], by using potassium chloride solution (pH 4.5) and sodium acetate buffer (pH 1.0). The absorbance was recorded at 520 and 700 nm in a UV-Visible spectrophotometer. Conventional and non-conventional solvent controls were performed in each determination to scan any possible interference. The total anthocyanin content was expressed as mg cyanidin-3-glucoside equivalents per liter (mg C-3-GE/L). Cyanidin-3-glucoside was obtained from Sigma Aldrich, St. Louis, USA and the salts from Cicarelli, Santa Fe, Argentina. The extraction yield of each solvent was determined as the amount of chemical component per L of extract.

#### 3.6.3. Total Alkaloids Content Determination

The alkaloid content of different extractive solutions (EW, DW, AW, NaDESs) was determined as described by Önal et al. [66]. The absorbance was recorded at 414 nm in a UV-Visible spectrophotometer. Apomorphine hydrochloride was used as standard (Merck, Darmstadt, Germany). Conventional and non-conventional solvent controls were performed in each determination to scan any possible interference. The experiment was performed in triplicate and the results were expressed as equivalent of apomorphine hydrochloride per milliliter (µg ACE/mL). The extraction yield of each solvent was determined as the amount of chemical component per mL of extract.

### 3.7. Antioxidant Activity Determination

#### 3.7.1. ABTS Free Radical Scavenging Activity

The antioxidant capacity assay was carried out by the improved ABTS^•+^ spectrophotometric method [67]. In this method, ABTS^•+^ solution (Sigma Aldrich, St. Louis, MO, USA) was mixed with different amounts of the extractive solution obtained from *N. tabacum* inflorescence powder. Conventional and non-conventional solvent controls were performed in each determination to scan any possible interference. Absorbance was recorded at 734 nm after 6 min. Results are expressed as SC_50_ values. SC_50_ (μg GAE/mL) was defined as the concentration of phenolic compounds necessary to scavenge the 50% of ABTS free radicals. Quercetin was used as a reference compound.

#### 3.7.2. Hydrogen Peroxide (H_2_O_2_) Scavenging

The H_2_O_2_ scavenging was assessed according to Fernando and Soysa [68]. The reaction mixture contained 12 mM phenol (Cicarelli, Origin in USA, packed in Santa Fe, Argentina), 0.5 mM 4-aminoantipyrine (Sigma Aldrich, St. Louis, USA), 0.7 mM H_2_O_2_ (Sigma Aldrich, St. Louis, USA), 84 mM sodium phosphate buffer (Cicarelli, Santa Fe, Argentina) at pH 7 and different concentrations of the extractive solution obtained from *N. tabacum* inflorescence powder. It was kept at 35 °C for 20 min. Then, horseradish peroxidase (0.1 U/mL, Sigma Aldrich, St. Louis, MO, USA) was added, and the mixture was incubated at 37 °C for 30 min. The absorbance was measured at 504 nm. Results are expressed as SC_50_ values in μg GAE/mL. Conventional and non-conventional solvent controls were performed in each determination to scan any possible interference. Quercetin was used as a reference compound.

### 3.8. Statistical Analysis

For the statistical analysis of the data, the Tukey test was applied, with a level of significance *p* < 0.05, by using the statistical package InfoStat V1.1 [69]. Main component analyses using R software 3.0.2 [70] were applied. Mean values were used for graphs, where each point on the graph corresponded to the mean value for each value. The heatmap with dendrogram using Euclidean distance and Ward’s clustering algorithm was applied to the standardized dataset using R software 3.0.2 [70].

## 4. Conclusions

To the best of our knowledge, this is the first work performed on the use of green solvents (NaDESs), an alternative solvent with lower environmental impact and toxicity than conventional solvents, for the recovering of valuable compounds, mainly polyphenols, from tobacco inflorescence, a pre-harvest waste of tobacco crops. The most promising results were obtained with acid-based NaDESs, mainly with SALA, that show more efficiency and selectivity to antioxidant polyphenol recovering from tobacco waste. The use of these tobacco waste extracts in cosmetic formulations seems promising. Further toxicological studies on these cosmetic ingredients from inflorescences such as tobacco pre-harvest waste are required to develop products aimed at promoting health care on a commercial scale. Therefore, NaDESs are an interesting alternative to organic solvents for the extraction of bioactives from waste to promote a circular economy for the tobacco industry.

The use of this source of metabolites can be of interest for the elaboration of cosmetic products. The morpho-anatomical botanical characterization of the plant material and the powders obtained from them was also carried out in order to allow their pharmacognostic identification in future products or as raw material, which could be commercialized in the form of dry inflorescences or as powders.

## Figures and Tables

**Table 1:** *Nicotina tabacum* var. Virginia. Dried, ground, and sieved inflorescence from tobacco.

Mesh Size (µm)	Weight after Sieving (g)	Yield in Percentage (%)
840	50.00	46.53
500	24.81	23.08
149	25.71	23.92
105	4.96	4.61
74	1.67	1.55
<74	0.30	0.27

**Table 2:** Natural deep eutectic solvents (NaDESs) used for extraction.

NaDESs Code	Components	Molar Ratio	pH
LAS	sucrose: lactic acid	1:4	4
SALA	sucrose: lactic acid: distilled water	1:5:7	1
CAP	propilen glycol: citric acid	4:1	5
FGS	fructose: glucose: sucrose: distilled water	1:1:1:11	6
CU	choline chloride: urea: distilled water	1:2:1.5	6

**Table 3:** Phenolic compounds, total flavonoids, total anthocyanins, and total alkaloids content of *Nicotiana tabacum* var. Virginia inflorescence powder extracts using conventional and non-conventional solvents.

*N. tabacum* Inflorescence Powder	TPC µg GAE/mL	TF µg QE/mL	TA µg ACE/mL	TAC mg C-3-GE/L
DW	1326.0 ± 7.6 ^a^	122.5 ± 8.8 ^d^	336.7 ± 2.9 ^c^	2.9 ± 0.3 ^b^
EW	2711.0 ± 11.5 ^c^	204.8 ± 5.1 ^e^	578.7 ± 5.6 ^e^	N/D
Methanol/HCl 1%	-	-	-	5.7 ± 0.0 ^c^
AW	2230.0 ± 4.0 ^b^	133.0 ± 2.3 ^d^	606.2 ± 1.8 ^e^	3.5 ± 0.4 ^b^
NaDESs				
LAS	2740.0 ± 5.5 ^c^	44.9 ± 6.1 ^b^	62.3 ± 0.4 ^b^	2.9 ± 0.1 ^b^
CAP	1305.0 ± 9.0 ^a^	14.4 ± 6.3 ^a^	30.0 ± 4.0 ^a^	1.5 ± 0.5 ^a^
FGS	2402.5 ± 9.7 ^b^	84.3 ± 4.9 ^c^	324.1 ± 3.2 ^c^	N/D
SALA *	3420.0 ± 9.4 ^d^	74.3 ± 5.5 ^c^	60.0 ± 4.4 ^b^	3.3 ± 0.9 ^b^
CU	2883.0 ± 9.7 ^c^	215.3 ± 3.2 ^e^	392.3 ± 8.0 ^d^	N/D

TPC: Total phenolic compounds; TF: total flavonoids; TA: Total alkaloids; TAC: total anthocyanins; GAE: Gallic acid equivalent; QE: quercetin equivalent; ACE: Apomorphine hydrochloride equivalent; C-3-GE: cyanidin-3-glucoside equivalents; DW: distilled water; EW: ethanol 70°; AW: Acetone: distilled water; NaDESs: LAS, CAP, FGS, SALA, CU. Values are reported as mean ± standard deviation of triplicates. Different letters in the same column indicated significant differences between extract according to Tukey’s test (*p* ≤ 0.05). * SALA: selective solvent for alkaloids.

**Table 4:** Antioxidant activity of *Nicotiana tabacum* var. Virginia inflorescence wastes powder extracts.

*N. tabacum* Inflorescence	ABTS (SC_50_ µg GAE/mL)	H_2_O_2_ (SC_50_ µg GAE/mL)
Conventional Extraction Solvents		
DW	12.0 ± 2.5 ^e^	37.0 ± 5.4 ^e^
EW	3.6 ± 0.9 ^b^	60.0 ± 6.6 ^h^
AW	8.0 ± 0.9 ^d^	42.0 ± 3.2 ^f^
Non-Conventional Extraction Solvents		
LAS	17.0 ± 2.4 ^f^	10.9 ± 2.8 ^b^
CAP	9.0 ± 1.8 ^d^	5.2 ± 0.9 ^a^
FGS	2.1 ± 0.8 ^a^	56.0 ± 9.3 ^g^
SALA	8.76 ± 1.2 ^d^	13.6 ± 5.1 ^c^
CU	5.7 ± 0.8 ^c^	45.0 ± 4.5 ^f^
Quercetin	1.40 ± 0.1 ^a^	17.3 ± 0.5 ^d^

GAE: gallic acid equivalent; QE: quercetin equivalent; ACE: equivalent apomorphine hydrochloride; GE: glucose equivalent; DW: distilled water; EW: ethanol 70°; AW: acetone: water; NaDESs: LAS, CAP, FGS, SALA, CU. Values are reported as mean ± standard deviation of triplicates. Different letters in the same column for each extract indicate significant differences according to Tukey’s test (*p* ≤ 0.05).

**Table 5:** Pearson’s correlation coefficients of ABTS and H_2_O_2_ scavenging versus TPC, TF, TA and TAC.

	Pearson’s Correlation Coefficients	
	ABTS	H_2_O_2_
TPC	−0.12	0.09
TF	−0.48 *	0.77 *
TA	−0.52 *	0.87 *
TAC	0.73 *	−0.58 *

* Correlation is significant at *p* < 0.05.

## Data Availability

Not applicable.

## Sample Availability

Samples are available from the authors.
